# Ambassador of People with Disabilities in the Workplace—Conducive Demographic and Professional Characteristics

**DOI:** 10.3390/ijerph17197036

**Published:** 2020-09-25

**Authors:** Urszula Załuska, Alicja Grześkowiak, Cyprian Kozyra, Dorota Kwiatkowska-Ciotucha

**Affiliations:** 1 Department of Logistics, Wroclaw University of Economics and Business, 53-345 Wrocław, Poland; urszula.zaluska@ue.wroc.pl; 2 Department of Econometrics and Operational Research, Wroclaw University of Economics and Business, 53-345 Wrocław, Poland; alicja.grzeskowiak@ue.wroc.pl; 3 Department of Statistics, Wroclaw University of Economics and Business, 53-345 Wrocław, Poland; cyprian.kozyra@ue.wroc.pl

**Keywords:** disability, attitudes towards people with disabilities, WHO ADS scale, statistical analysis of survey results, disability in the workplace

## Abstract

This paper introduces into the analysis the concept of the ambassador of people with disability in the workplace. A kind and friendly person in the workplace, who creates a positive atmosphere around people with disabilities, may play a crucial role in their adaptation on the open labor market. Presence of such a person is especially important in entities that did not previously employ people with disabilities. It is vital that employers who would like to employ people with disability possess knowledge about demographic and professional characteristics that predispose employees to perform this special role. On the one hand, in this article we attempted to evaluate the differentiation in the perception of the issue of disability due to demographic and professional characteristics of respondents, and, on the other hand, to identify features that favor being an “ambassador of people with disabilities” in the workplace. The study was conducted in 2019 on the representative samples of Internet users from 8 European countries using Computer-Assisted Internet Interviews. For the purposes of the study, we used the Attitudes to Disability Scale WHOQOL Group test and a proprietary questionnaire. As for the methods of analysis, we relied on the classical analysis of variance and logistic regression. The conducted study showed that the perception of the issue of disability is significantly related to demographic and professional characteristics of respondents, and that the role of the ambassador is the most appropriate for a middle-aged woman with a good knowledge of disability issues, indecisive in the workplace.

## 1. Introduction

The full inclusion of people with disabilities means that all of them are provided with the same conditions to function in society, including the right to make decisions about their lives, the possibility of starting a family or non-discriminatory access to workplaces. This is stated, among others, in the provisions of the Convention on the Rights of Persons with Disabilities [1] adopted in 2006 by the United Nations and ratified by all European Union countries. Unfortunately, very often these provisions are not respected in practice, since the employment rate for this group of people is still very low [2,3]. According to the results of the most recent comprehensive European research on the situation of people with disabilities, in most European countries the difference between the employment rate of people with disabilities aged 15–64 and those without disabilities was 20 percentage points [4]. The differences at the level of 20–40 percentage points to the disadvantage of people with disabilities are also indicated in the latest OECD report dedicated to groups which find themselves in an unfavorable situation on the labor market [5]. Moreover, in many European countries, especially in post-communist ones, people with disabilities working according to their level of education are still a rare phenomenon [6,7]. The same is true of the possibility of full functioning in the public space for this group of people [8]. This situation is largely influenced by attitudes towards people with disabilities presented in mass culture which may promote prejudices and stereotypes towards this group or openness and the right to normality for everyone. Semiotic research is very well suited to the analysis of content in public discourse, in which existing texts are analyzed, i.e., both written text and images, signs, symbols, films, commercials or reportages [9]. In semiotic research, the concept of Greimas’s semiotic square could be used, according to which the meaning and value in a specific culture are generated by a place in the structure formed by the relations of binary opposites (e.g., good—bad) and the contradictory relations that complement them (e.g., not-bad—not-good) [10,11].

An example of a semiotic study relating to the perception of people with disabilities in popular culture is the study that was conducted at our request in 2018 by a company specialized in this type of research to evaluate the situation in Poland [7]. The subject of the study covered cultural texts that mainly came from Poland, as well as from other countries. The company analyzed materials mostly from 2016–2018, including over 3000 press releases, about 100 films, 700 texts from the Internet, and several dozen Polish and foreign advertisements. According to the results of the semiotic research, in Polish mass culture a person with a disability is mainly portrayed as a hero struggling with everyday functioning despite their disability/handicap; fighting for a better life; or being an excluded, weak person, in need of help from others, dependent on the support of the state and their loved ones. To a very small extent and slowly, under the influence of cultural changes, we can notice people with disabilities in the public space, aware of their rights and possibilities, treating disability as a feature distinguishing them from others. In Polish public discourse, disability is rarely associated with normal family and professional life [7].

The results of the semiotic research in relation to Polish mass culture inspired us to check the differentiation in the perception of the issue of disability due to demographic and professional characteristics of respondents in an international study on the representative samples of respondents from eight European countries. In the literature on the subject, there are many examples of scientific research on attitudes towards people with disabilities and methods of measuring them [12,13,14]. In studies in which the importance of demographic and professional characteristics for the perception of disability was analyzed [15,16], it was found, among others, that men represent more discriminatory attitudes towards people with disabilities than women; women appear to have more understanding about the need to adapt working conditions to the needs of people with disabilities, whereas better educated and younger participants respond to people with disabilities better than less educated and elderly ones. Previous studies indicated that elderly people assess the acceptance of motor and sensory disability in the workplace definitely better than the representatives of younger generations, just like people with higher education compared to those less educated. Young people, on the other hand, perceive the acceptance of mental disorders in a definitely more positive way than older employees [17].

We used two sources of information to check the possible differentiation in the perception of the issue of disability due to demographic and professional characteristics of respondents. The first one was the Attitudes to Disability Scale (ADS) test, widely used to measure the perception of people with disabilities in everyday life, developed by a team led by M. Power (The World Health Organization Quality of Life Group—WHOQOL Group) [18,19,20]. The test consists of sixteen statements relating to the perception of the functioning of people with disabilities in society, grouped in four main areas: Inclusion (items 1–4), Discrimination (items 5–8), Gains (items 9–12), Prospects (items 13–16); see Appendix A, Table A1. The respondent expresses their beliefs on the 5-point Likert scale, where 1 means “totally disagree”, whereas 5 meant “completely agree”. The items of the ADS scale are formulated as negative statements about people with disabilities. Factor 3 (Gains) is an exception, in which items indicate positive features related to disability.

The second source of information was a proprietary questionnaire in which respondents were asked, among others, the questions in Table 1, about the correctness of the existing systemic solutions regarding the policy towards people with disabilities, the atmosphere of social openness towards people with disabilities, and employment privileges for people with disabilities, as well as the employers’ knowledge of disability issues. Respondents were answering questions using the 4-point scale, where 1 meant “Definitely no” whereas 4 meant “Definitely yes”.

Additionally, in order to assess the significance of respondents’ characteristics for particular openness towards people with disabilities in the workplace, we used one of the questions from the ADS test and a question from the proprietary questionnaire (see Appendix A, Table A2).

Two research questions were formulated:

The first research question: Does the perception of the of functioning of people with disabilities in society differ due to demographic and professional characteristics and respondent’s knowledge of the issues? In other words: Do the representatives of different groups perceive the striving for normality in the functioning of people with disabilities differently, and do these differences result more from demographic or professional characteristics, or the level of knowledge of the subject?

The second research question: Is it possible to indicate demographic and professional characteristics and the elements of respondent’s knowledge of the subject which contribute to a much greater openness towards people with disabilities in the workplace? In other words: Is it possible to appoint an ambassador of people with disabilities in the workplace? This is particularly important when planning to employ people with disabilities in an entity where such people have not worked so far.

## 2. Materials and Methods

### 2.1. Methods of Data Analysis

In order to find the answer to the first research question, we relied on the classical analysis of variance [21,22,23] using F statistic, including tests of significant differences between the group means. Using these methods, it is possible to determine the presence/absence of statistically significant differences in the perception of disability between groups of people differentiated on the basis of specific characteristics. Due to the interdependencies between demographic and professional characteristics, in addition to the univariate analysis of variance, multi-way analysis of variance models [21] was used but only of the main effects, without interactions.

In order to find the answer to the second research question, we used the results obtained from the methods applied to achieve the first objective. While creating the profile of the ambassador of people with disabilities in the workplace, we assessed the most important features. The criterion for categorizing someone as an ambassador was constructed by combining extreme answers to two survey questions as a binary feature (see Appendix A, Table A3—grey field). It was assumed that only a person who strongly rejects the lack of prospects for people with disabilities and who is firmly convinced about the possibility of compensating for disability in the workplace meets the expectations of the ambassador for disability issues at work. Then, using logistic regression [22,23,24], it was assessed which of the characteristics were most important for the chance of meeting this criterion.

In the analysis of the survey questions we have used two approaches: the analysis of exact answers and a psychometric approach. As part of the research on exact answers, we analyzed the selection of a specific category of answers in the survey question, e.g., a declaration of very good knowledge of the issues of people with disabilities. As part of the psychometric approach [21], on the basis of the analysis of the correlation between the variables, we evaluated their unidimensionality by factor analysis and reliability using the Cronbach’s alpha coefficient. Psychometric analyses of the ADS scale were presented in the previous article [25]. In all four areas, the obtained values of reliability coefficients were similar and satisfactory, which allowed the use of the summative scale. In this paper we focused on the general situation assessment scale. If the measurement reliability criteria using the summative scale were met, the analysis was performed using the mean of the variables included in the scale.

The analysis of variance was performed both as one-way and multi-way of main effects without interactions. Multivariate ANOVA models were created for each dependent variable using the forward stepwise method to verify the possible impact of interrelationships between the categorical variables. The significance obtained in the multivariate model was presented in tabular form, and the mean values for the categories with confidence intervals obtained in the one-way analysis of variance were presented graphically. In the case of characteristics possessing more than one category, the Tukey HSD (honest significant difference) post-hoc test for unequal sample sizes [22,26] was additionally used to identify those categories between which there were significant differences.

In order to assess the importance of characteristics of respondents that predispose them to be ambassadors of people with disabilities in the workplace, we used logistic regression. We also relied on the forward stepwise method and the likelihood ratio tests [23]. In this method, testing the introduction of variables into the model was based on the significance of parameter estimate statistics, whereas testing their removal—on the probability of likelihood-ratio statistics based on maximum partial likelihood estimates. It was assumed that the variable was introduced into the model if the probability of its estimate statistics was less than 0.05 and removed if the probability was higher than 0.10. The quality of the model was evaluated on the basis of the collective test of model coefficients and the Hosmer–Lemeshow test [27]. When assessing the significance of the influence of independent variables on the dependent one, we used Wald statistics [23], whereas in all statistical analyses, we relied on the Statistica 12.5 (StatSoft, Inc., Tulsa, OK, USA) and IBM SPSS 25 software (IBM, Armonk, NY, USA).

### 2.2. Data Collection and Research Sample

A comparative research on representative samples of Internet users aged 18 to the official retirement age from eight European countries was conducted in September–December 2019 using computer-assisted Internet interviews (CAWI). It involved at least 500 people from Belgium (*N* = 521), Bulgaria (*N* = 525), Greece (*N* = 519), Spain (*N* = 520), Germany (*N* = 536), Poland (*N* = 528), Sweden (*N* = 524) and Great Britain (*N* = 536), and detailed information about it can be found in [25]. At the country level, the sample was of random-quota nature, and it was representative due to features such as gender, age, education, place of residence and region of residence. Quotas were determined on the basis of the population structure in individual countries, and in order to do that, the data from the Eurostat database for 2018 were used. During the implementation of the research, the highest randomness of recruited respondents was ensured by using various ways of reaching them, including Internet panels operating in a given country and mobile applications for telephone/tablet (dynamic sampling). The respondents were informed about participation in CAWI research. In this article, we concentrate on the differences obtained in the results for people with different demographic and professional characteristics for questions about the social perception of the issue of disability—personal feelings and opinions relating to the assessment of the general situation.

During the study, 4827 complete questionnaires were collected, of which 4209 respondents were selected who constituted the main sample corresponding to the assumed quota structure for each country. For this sample, we carried out analyses, the results of which are discussed in the article. The characteristics of respondents according to the features taken into account in the study are presented in Table 2.

## 3. Results

### 3.1. Factor Analysis and Measurement Reliability

Presented in Table 1, the set of questions concerning the assessment of systemic solutions, the atmosphere of social openness, employee privileges and the employers’ awareness of problems when employing people with disabilities were analyzed with the help of factor analysis using the principal components to extract factors and varimax rotation thanks to which it is possible to obtain more informative interpretation of the factors [21,28]. The obtained results are presented in Table 3.

The results of the factor analysis clearly show a two-factor structure, which explains almost 75% of the overall variability. All variables, except for the assessment of the need for special privileges (benefits) in the workplace for employees with disabilities, are strongly correlated with each other. This justifies the development of a summative scale consisting of assessments of state policy (item 1), social atmosphere (item 2) and the knowledge of employers (item 4). This scale will be referred to as “Situation” later in this article. The reliability of the scale obtained in this way is sufficiently high [21], and the Cronbach’s alpha coefficient is 0.74. Question 3 concerning the legitimacy of special privileges (benefits) for employees with disabilities should be treated separately in the analysis as not correlated with the others (hereinafter: Privileges).

### 3.2. Analysis of Variance

Analysis of variance was used to assess the influence of selected respondents’ features on the investigated predicted characteristics. These were the opinions formulated within the four dimensions of the ADS and the Situation assessment scale. Question 3 from Table 1 about the legitimacy of privileges (benefits) for people with disabilities at work was analyzed separately. In terms of attitudes towards people with disabilities and overall situation assessment, the questions were formulated in such a way that the set of the-larger-the-better variables covered Gains and Situation and the set of the-smaller-the-better variables, Inclusion, Discrimination and Prospects. Statements in the area of Gains are positive, indicating that disability can make someone stronger, smarter, more determined and more successful. Higher evaluations for the Situation scale mean that, according to respondents, there is a more effective inclusion policy applied, a better social atmosphere of understanding the needs and possibilities of people with disabilities, and that employers’ knowledge concerning employing someone with disability and the organization of their work is rated in a more positive way. Statements in the areas of Inclusion, Discrimination and Prospects, on the other hand, were negative, indicating that i.e., people with disabilities have problems with social involvement, that they are a burden for the family or society, that they are vulnerable to take advantage of, or that they should have lower expectations than other people. The Privileges variable is not of explicit and unambiguous nature. On the one hand, due to the special needs of people with disabilities, one should take into account such privileges (e.g., additional days of leave for rehabilitation necessary for everyday functioning). On the other hand, too many privileges prevent the pursuit of normality when employing representatives of this target group. It is also worth emphasizing that employee privileges often evoke a lot of negative emotions among other employees, especially those with a low level of knowledge about disability and consequences, problems and needs resulting from it.

As for the independent variables, the following dichotomous features were used: sex, age (divided into groups of 18–39 and 40–65-year-olds), place of residence (countryside vs. city or town), declaration of knowledge of disability issues (a lot of knowledge vs. other answers), decision-making as regards employing people at the workplace (main decision maker vs. other people). As an independent variable, we used a three-category one representing the declared involvement or experience with disability: (1) people personally involved are people with disabilities and those professionally taking care of them; (2) people who have experienced disability are usually those who have people with disabilities in their families, neighborhoods, at their workplace or among friends and (3) respondents declaring no experiences with people with disability. The division into dichotomous features and three-category ones was the result of previous analyses based on the assessment of the significance of difference between means in the opinion of respondents from individual groups distinguished in the questionnaire.

The results of the study of the significance of the influence of categorical features on the features in the multivariate analysis of variance model, only for the main effects [21], are shown in Table 4.

The characteristics that differentiated the opinions of respondents most frequently appeared to be sex, age and experience with disability. As for these features, significant differences occurred for five out of the six investigated dependent variables, and it is worth emphasizing that in the vast majority of cases, the *p* value was below 0.01. The least differentiating characteristic is the size of place of residence, for which significant differences were found only in the case of half of the dependent variables. Taking into account the differentiation within the considered evaluation areas, the most common significant differences occurred for the Inclusion dimension and the Situation scale. Significantly convergent results were obtained for the one-way analysis of variance, which leads to the conclusion that the influence of the distinguished characteristics on the dependent variables was much stronger than their mutual relations, which were initially assumed.

**Figure 1:**
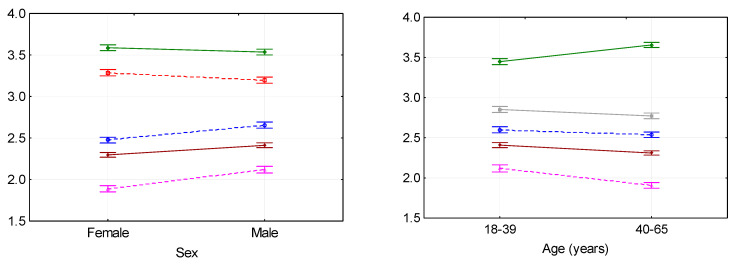

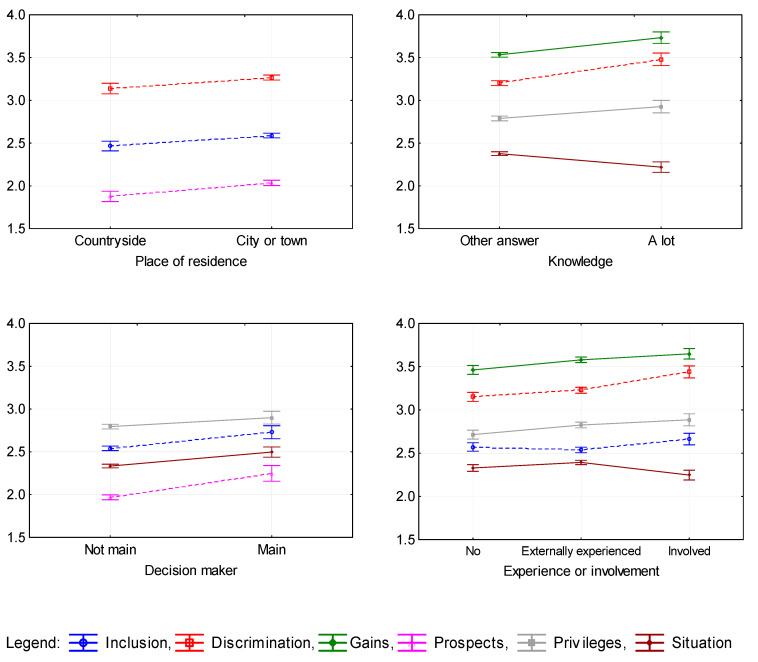
Graph of means of categorical variables with 95% confidence intervals.

Figure 1 shows the averages evaluations estimated for the categories of independent variables with 95% confidence intervals obtained in the one-way analysis of variance for those categories for which the influence of the categorical feature on the dependent variable appeared to be significant. For the characteristics that did not significantly differentiate the selected sub-populations, the graphic visualization was omitted. The smaller-the-better characteristics have a different pattern—they are presented with dotted lines, which is important from the point of view of interpretation of the obtained results. The interpretation will be made below for each categorical feature individually.

#### 3.2.1. Sex

Women evaluate the situation of people with disabilities better than men in terms of the possibility of their social inclusion and prospects, as well as the benefits that may result from disability. At the same time, they notice greater discrimination against people with disabilities and assess the overall situation in terms of inclusion policy, social atmosphere and the preparation of employers for employing people with disabilities in a more negative way.

#### 3.2.2. Age

People from older age groups, above all, emphasize the fact that disability can make someone stronger and more determined, and they evaluate the prospects of people with disabilities and the possibility of their social inclusion better than the representatives of the younger age group. On the other hand, they perceive the general situation worse, and they are less in favor of special employment privileges for people with disabilities.

#### 3.2.3. Place of Residence

As for the place of residence, respondents’ opinions differed significantly for the three areas of the ADS scale in which the statements were negative. People who live in the countryside perceive the situation of people with disabilities in all those areas in a more positive way, just like the possibilities of social inclusion and prospects. They also notice the manifestations of discrimination to a lesser extent.

#### 3.2.4. Knowledge of Issues Related to Disability

People declaring a very good knowledge of the issue of disability, compared to other respondents, evaluate the general situation in a less positive way, especially in terms of the effectiveness of the policy enabling full inclusion of people with disabilities and the social atmosphere of understanding the needs and possibilities of this group. They seem to notice discriminatory behavior in society and benefits that may result from disability to a greater extent. They are more frequently in favor of special privileges (benefits) for people with disabilities in the workplace.

#### 3.2.5. Decision-Making in Hiring Employees

In the study, we compared a group of people responsible for such decisions to other respondents. Decision-makers evaluate the possibilities of inclusion of people with disabilities as well as their prospects in a more negative way. However, higher average evaluations were noted in the case of opinions about the general situation, especially when assessing the knowledge of employers concerning the employment of people with disabilities and organizing their work. The last significant difference concerns special employee privileges for people with disabilities. In this case, decision-makers notice such a need more frequently than other respondents.

#### 3.2.6. Experience and Involvement in Disability

In the case of inclusion, we noted a significant difference in the opinions between people with external experience with disability and those personally involved in such issues, where the latter perceive the possibility of social inclusion of people with disabilities to a greater extent. This group also emphasizes the issue of discrimination more explicitly than the other two groups. The benefits resulting from disability were assessed in a significantly less positive way by people who have no experience with disability compared to the rest of respondents. While assessing the general situation in the country, the opinions of respondents with external experience and those involved in disability differed significantly—the general situation was evaluated worse by people suffering from disability and by those professionally involved in this issue. As for assessing the need for privileges for people with disabilities in the workplace, there were significant differences between people who had no experience with disability and other groups—the former notice such a need less frequently.

### 3.3. Logistic Regression

The dependent variable was constructed as a binary feature on the basis of respondents’ answers to two questions in Table A2 (Appendix A). The first question concerned the opinion on the possibility of effective compensation for limitations resulting from disability by an appropriate workplace or its equipment (a question from the proprietary questionnaire). The second one concerned the assessment of the statement saying that people should not expect too much from the people with disability (a question 14 from the ADS test). Assigning a given respondent to the category of ambassadors of people with disabilities in the workplace depended on the answers that they gave to these two questions. We assumed that an appropriate candidate for the ambassador should be convinced of the possibility of effective inclusion and the use of the potential of people with disabilities in the workplace. Taking into account the specificity of the analyzed questions, it should be a person who shares the opinion about the possibility of effective compensation for limitations resulting from disability by providing an appropriate workstation. At the same time, he or she should reject the belief that one shall not expect too much from people with disabilities. Ultimately, respondents assigned to the group of ambassadors were those who answered “I completely agree” in the first question and “I totally disagree” in the second one (see Table A3, Appendix A—grey area). This condition was met by 22.05% of all respondents. They were assigned the value of a binary feature equal to 1—an appropriate candidate for the ambassador—while the remaining respondents were assigned the value of zero. Potential independent variables describing demographic and professional characteristics of respondents and their knowledge of disability issues along with the assigned values for each category of binary features are presented in Table 5. In addition to binary features, the model includes a quantitative Age variable.

Before multivariate logistic regression model was estimated, dependence between predicted Ambassador variable (defined as in Appendix A, Table A2) and every separated binary variable was investigated by means of chi-squared χ^2^ independence test in 2 × 2 cross-tables [21]. Results are shown in Table 6. Binary age categorical variable is also included although quantitative variable age (in years) was used in logistic regression model.

All characteristics, except for the place of residence, are significantly related to the Ambassador variable. The strongest relationship is for age, since over 26% of respondents aged 40–65 qualify for the ambassador category, whereas for the younger group this amount drops to 17%.

Ultimately, five independent variables were included in the multivariate logistic regression model which were chosen by means of forward stepwise selection. In order of inclusion, these are age, sex, experience with disabilities, knowledge of the issues of people with disabilities and decision-making in the workplace in the area of employment. It means that these five variables significantly differentiate the population in terms of the features of a suitable candidate for the ambassador of people with disabilities in the workplace. Two features were not included in the model, the place of residence and involvement in disability, due to the lack of statistically significant and sufficiently strong differentiating properties. The model meets the assumptions of a good logistic regression model and correctly predicts the category of the dependent variable as proved by the result of the collective test of the model coefficients (Chi-square = 7.032, *p* = 0.008). On the basis of the Hosmer–Lemeshow test [27], the hypothesis concerning the equality of the observed and predicted values on the basis of the model was positively verified (there is no basis to reject the null hypothesis), which allows us to assume that the model is well suited to the empirical data. The results obtained for the individual variables included in the regression model are presented in Table 7.

All the variables included in the model significantly influence the values of the dependent variable. This is proved by the Wald statistics and the *p*-value corresponding to these statistics.

The variables most strongly differentiating the analyzed community in terms of being an ambassador of a person with disability in the workplace are demographic characteristics, namely, age and sex. Being one year older, a respondent increases his or her chances of being a suitable candidate for an ambassador almost 1.025×. In the case of sex, compared to women, men are 0.7× less likely to admit that the effective employment of people with disabilities may be the result of appropriate adaptation of the workplace and lack of assumption of the necessity of creating lower requirements for people with disability. Another variable included in the model was experience with disability understood as contact with people with disabilities in various relationships (family, neighborhood, work and friends), being a person with a disability or being professionally related to this issue. People with such experience are predestined to the role of an ambassador with a chance of over 1.37× greater than those without it. At this point, it is worth emphasizing that respondents who have experience with people suffering from disabilities appreciate their possibilities in the workplace. This means that the lack of openness is mainly due to the lack of experience and, consequently, a lack of knowledge and not primarily the result of limitations connected with disability. People who declare a very good knowledge of the issues related to disability have 1.314× greater chance of becoming a suitable candidate for the ambassador of people with disabilities in the workplace than people without an adequate knowledge in this area. The last important characteristic included in the model was decision-making in the workplace concerning hiring employees. It is worth noting that those who can make such decisions are more skeptical about them than people who do not have such authority. This is probably connected with the level of responsibility for the functioning of a given entity which a respondent has.

### 3.4. Comparison of Perceptions between Ambassadors and Non-Ambassadors

At the end of the analyses, we made a comparison of how belonging or not belonging to the group of ambassadors translates into features measured with the ADS scale and the questions presented in Table 1. The comparison was performed using *t*-test with separate variance estimates (Welch modification of Student’s test). The results are presented in Table 8.

There is no significant difference between ambassadors and non-ambassadors when it comes to the perception of discrimination and the legitimacy of ensuring privileges for people with disabilities in the workplace. As for the remaining characteristics, the observed significant differences show, as expected, that ambassadors are definitely more sympathetic towards people with disabilities. Ambassadors, more often than non-ambassadors, notice the possibility of obtaining gains despite disability. However, they less often agree with the lack of inclusion in society and the lack of prospects. They also evaluate the social situation in a less positive way, which may motivate them even more to support people with disabilities.

## 4. Discussion

The conducted research shows that the perception of the issue of disability is significantly related to demographic and professional characteristics of respondents. It is worth noting that sex is a strongly differentiating factor. Women are more open to people with disabilities than men, but at the same time they are more critical of external conditions such as inclusion policy, prevailing social atmosphere and the level of employers’ preparation. The obtained results confirm the previous findings of other authors. The phenomenon of women displaying less discriminatory attitudes towards people with disabilities than men has been described in the literature. In one of their studies, Popovich et al. [16] stated that women display more positive reactions towards a co-worker with disability. In another research, they did not find any significant relationships according to gender, but clearly indicated that women to a greater extent recognize the need to adapt the workplace to the needs of people with disabilities. Gordon et al. [29], while analyzing attitudes towards people with mental illness/ retardation, found that women have greater knowledge of mental illness and feel more comfortable than men when interacting with people with this type of disability. Tervo et al. [30] evaluated the attitudes of first-year students of medicine towards people with disabilities using various measurement scales and found that male students showed worse attitudes than female ones. Hergenrather and Rhodes [31] studied the influence of social context on attitudes towards people with disabilities and identified three factors reflecting contextual subscales (dating, marriage and work), for which women scored higher. It is worth pointing out that the results of some studies in this area are not unambiguous. McLaughlin et al. [32] found that women are less likely to formulate discriminatory employment judgments about co-workers with disabilities than men, but at the same time, they could not find any differences relating to gender when it comes to attitudes towards those people and their approach to accommodations, although it is necessary to note that the results of some research in this field are not unambiguous [32]. Differences related to sex may result from greater pragmatism in men and stronger emotional attitudes in women, including greater empathy [33], or the fact that the latter more often take on the roles of caregivers.

Age is another important demographic factor. The results of our research show that as the age increases, so does the openness towards people with disabilities and the assessment of the possibility of their social inclusion. Such results are in contradiction with the findings of other researchers who indicated that younger people are more likely to develop favorable attitudes towards people with disability. Strohmer et al. [34] verified attitudes towards people with selected types of disability in relation to situations such as work, dating and marriage and found that younger age is a variable strongly influencing positive results measured on a disability social relationship scale. A similar conclusion was presented by Goreczny et al. [35] after conducting a study on individuals attending a conference concerning the quality of life of persons with disabilities. In the review of the literature, Vornholt et al. [15] stated that if the research presented by other authors confirms the disparity in acceptance of people with disabilities at work depending on age, younger people show more positive attitudes than older ones. The different results found in our study, i.e., the positive attitude displayed by older research participants can be explained by their life experience, the possibilities of contact with people with disabilities and greater tolerance determined by their own weaknesses. However, it is debatable whether the greater openness of young people displayed in other studies is presented only on a declarative level, or whether it is put into practice. What leads to such conclusions are, among others, the results of CATI research on representative samples of Polish employees and employers carried out by the authors of the article in 2019 [7].

The obtained results allow us to conclude that the perception of disability is conditioned by knowledge and experience gained in this area. The declaration of a very good knowledge of disability issues is accompanied by a poorer assessment of inclusion policy, social atmosphere and the degree of discrimination. In turn, more emphasis is placed on benefits that may result from disability and opting for the existence of privileges in the workplace. Interestingly, having experience with disability also means that people display greater acceptance of those privileges. However, the group of people most involved in the issue of disability better perceives the possibilities of social inclusion, notices discrimination to a greater extent and is more critical of the situation in the country. The conducted study confirms the results obtained by other researchers who identified a positive correlation between favorable attitudes towards people with disabilities and having experience in this field. Barr and Bracchitta [36], while analyzing the attitudes of students, found that a positive attitude is strongly associated with the fact of having contact with friends with disabilities and participating in activities together with people with disabilities. Huskin et al. [37] checked the perception of 10 types of disability and concluded that regular contact resulted in smaller social distance, and the contact experience itself was a significant factor for 6 types of disability. Unger [38] considered the significance of experience in the context of readiness to employ a person with disability, concluding that previous experiences with employees with disabilities resulted in a more favorable perception. McDonnall et al. [39], when analyzing the attitudes of employers towards people who are blind or visually impaired, found that experience in employing such people as well as knowledge are of key importance. Daruwalla and Darcy [40] stated that contact with people with disabilities was more likely to change one’s attitude towards disability than providing information about it. Kleynhans and Kotzé [41] studied the attitudes of co-workers towards people with physical disabilities and proved that contact with them, as well as knowledge, can have influence on shaping these attitudes. Chen et al. [42], having studied Hispanic small business owners, concluded that the key factors determining employment included having a family member or friend with disability. The issue of perceiving people with disabilities by employers was also raised in [43], presenting a similar conclusion—positive attitudes are associated with greater experience with people with disabilities in the workplace. It is worth emphasizing that literature sources indicate that experience itself may be insufficient and what is also important is its quality [44].

Another aspect that influences the perception of disability is the position of people in the occupational hierarchy. Decision-makers evaluate the possibility of inclusion and the prospects of people with disability in a less positive way. Additionally, they are less willing to take risks related to, for example, financing activities aimed at preparing a workplace for the needs of people with disabilities, especially in the absence of experience in employing such people, assessing their work, and values for the organization. These findings are in line with results presented by other authors. The review of the literature [14] shows that despite generally positive employers’ attitudes towards people with disabilities, the practice of employing them may display some signs of discrimination. Barriers on the part of employers may be related to beliefs that people with disabilities do not do their jobs properly, concerns about a drop in income, legal disputes and the reactions of colleagues [45].

The obtained results seem to be valuable for the area of human resource management in economic entities. They allow of the initial selection of people recommended to support employees with disabilities in the workplace, which should significantly increase the effectiveness of professional adaptation of this group. However, it is worth emphasizing that the conducted analyses are associated with certain limitations. All statements provided by respondents are declarative, and the nature of the study did not allow a confrontation between the views presented and practical actions.

The research results presented in the article are part of a larger project dedicated to increasing the motivation of employers to employ people with disabilities on the labor market. The Go4Diversity Project financed by the European Social Fund and conducted within the Polish–Belgian–Swedish partnership assumed, among others, numerous qualitative studies, the results of which were omitted due to the objective of the study. Individual in-depth interviews (IDI) conducted in Poland with respondents employing people with disabilities on the labor market showed that an ambassador who introduces a person with disability to the team and improves their image may be of fundamental importance to reducing the level of stress, shortening the adaptation process in the workplace and increasing the probability of success. Similar aspects were mentioned by people with disabilities themselves. It is worth noting that the “ambassador of people with disabilities in the workplace” significantly reduces the risk of being rejected or disapproved by other team members (co-workers). As the research results show, it is “co-workers” that are often a bigger barrier to employing people with disabilities than the lack of openness towards this group on the part of employers [7,46,47]. One of the main reasons for cold reception on the part of able-bodied team members can be the “privileges” meant for people with disabilities existing in each country, which may vary greatly due to legislation, but which often cause problems and make full inclusion impossible. Hence our research on finding the characteristics that would allow us to define the profile of a person who could best carry out this diplomatic mission in the workplace. We are aware that the concept of the “ambassador of people with disabilities in the workplace” requires further research, especially in the area of real changes in practice.

## 5. Conclusions

In the article we analyzed the impact of selected demographic, professional and experience-related characteristics on the social perception of people with disabilities and the assessment of the situation in the country in terms of openness to people with disabilities. The characteristics that differentiate opinions most strongly in this respect are age, sex and experience in the area of disability, whereas the place of residence seems to have the least influence. Using logistic regression analysis, we managed to identify the characteristics of the ambassador of people with disabilities in the workplace—a person displaying greater openness towards people with disabilities and conviction of the possibilities of using their potential. We have also distinguished five characteristics important from this point of view, which has not only a cognitive aspect, but also practical significance for management, especially in entities that are about to decide to employ people with disabilities and have had no such experience before. In the studies conducted so far, differences between specific countries have not been taken into account. This particularly refers to cultural differences that may influence and bias the results of research carried out on international samples. This issue is so important that it deserves a separate publication, and we decided to focus on this aspect in our subsequent research. Nevertheless, the aim of our article was to show possible differences between the answers given by respondents with various demographic or professional characteristics, regardless of the cultural differences of the countries they come from. It is worth noting that the differences in the answers of female and male respondents, regardless of the country of origin, are also indicated in research carried out, among others, by G. Hofstede, who stated that the answers given to the same questions by men and women were regularly different [48]. In subsequent studies, it would be interesting to analyze the significance of the identified characteristics of the ambassador in each country separately.

## Figures and Tables

**Table 1:** Analyzed questions from the proprietary questionnaire.

No	Question
1	In your opinion, does your country carry out an effective policy that allows for full integration of the people with disabilities?
2	In your opinion, is there social atmosphere of understanding the needs and possibilities of people with disabilities in your country?
3	Do you think that the people with disabilities who have a job should have special employee privileges, for example, a shorter working day, longer holidays, etc., in your country?
4	How do you think employers in your country get sufficient knowledge on how to employ a person with disabilities and organize his/her work?

**Table 2:** Research sample—structured according to selected demographic and professional characteristics and knowledge of disability issues (*N* = 4209).

Characteristic	Characteristic Category	Percentage of Respondents (%)
Sex	Female	49.9
	Male	50.1
Age	18–34 years old	32.4
	35–49 years old	33.6
	50–65 years old	34
Size of the place of residence	Countryside	19.6
	City up to 50 k residents	30.7
	City from 50 k to 200 k residents	22.2
	City from 201 k to 500 k residents	11.6
	City over 500 k residents	15.9
Decision maker as regards employing people ^1^	Main decision maker	21.8
	Make decisions together with the other people	23.2
	Little influence on the decision	14.7
	Not make decisions on employing other people	40.3
Knowledge about the problems of people with disabilities	A lot	14.4
	Much	27.7
	A little	43.6
	I do not know anything about their problems	8.8
	Difficult to say	5.5
Experience in contacts with people with disabilities ^2^	Person with a disability in my family	23.4
	Person with a disability among my friends	28.4
	Person with a disability in my neighborhood	22.5
	Person with a disability at my workplace	14.7
	I am a person with a disability	10.4
	I professionally take care of people with disabilities	5.5
	I have no experience with people with disabilities	24.1

^1^ Only among employees or working in own company, farm (*N* = 2458). ^2^ Multiple answers possible.

**Table 3:** Factor loadings after varimax rotation.

Question Number	Factor 1	Factor 2
1	0.850	−0.007
2	0.840	−0.009
3	0.023	0.998
4	0.748	0.069
Explained variance	1.989	1.002
Share	49.7%	25.0%

**Table 4:** Significance of predictors in multi-way ANOVA main effects only model (F test).

	Predicted	Inclusion	Discrimination	Gains	Prospects	Situation	Privileges
Categorical							
Sex		***	***	*	***	***	ns
Age		*	ns	***	***	***	**
Size of the place of residence		***	***	ns	***	ns	ns
Knowledge		ns	***	***	ns	***	**
Decision maker		***	ns	ns	***	***	*
Experience or involvement		***	***	**	ns	**	**

*** *p* < 0.001, ** 0.001 ≤ *p* < 0.01, * 0.01 ≤ *p* < 0.05, ns—non-significant.

**Table 5:** Potential binary independent variables in logistic regression.

Variable	Category	Value	Variable	Category	Value
Sex	Female	0	Decision maker	No	0
	Male	1		Yes	1
Place of residence	Countryside	0	Experience with disability ^1^	No	0
	City	1		Yes	1
Knowledge of disability issues	Other answers	0	Involvement in disability ^2^	No	0
	Very good	1		Yes	1

^1^ A binary feature obtained from the categorical variable 3, Experience or involvement, where 0 means *I have no experience with people with disabilities*, whereas 1 means the remaining answers. ^2^ A binary feature obtained from the 3-categorical variable Experience or involvement, where 1 means *I am a person with a disability* or *I professionally take care of people with disabilities*, whereas 0–remaining answers.

**Table 6:** Significance of dependence between Ambassador feature and independent variables (χ^2^ test).

Variable	*p*-Value	Group 1	Percentage of Ambassadors in Group 1	Group 2	Percentage of Ambassadors in Group 2
Sex	***	Female	25.3%	Male	18.9%
Age	***	18–39	17.1%	40–65	26.2%
Size of the place of residence	ns	Countryside	22.7%	City or town	21.9%
Knowledge	***	A lot	27.6%	Other answer	21.1%
Decision maker	**	Main	17.1%	Not main	22.8%
Experience with disability	***	No	17.9%	Experienced	23.4%
Involvement in disability	*	No	21.4%	Involved	25.5%

*** *p* < 0.001, ** 0.001 ≤ *p* < 0.01, * 0.01 ≤ *p* < 0.05, ns—non-significant.

**Table 7:** Results of multivariate logistic regression—variables in the model.

Step	Variable	*B*	Standard Error	Wald	df	*p*-Value	Exp(*B*)
1	Age	0.024	0.003	68.072	1	0.000	1.024
2	Sex	−0.371	0.076	23.691	1	0.000	0.690
3	Experience with disability	0.318	0.095	11.217	1	0.001	1.374
4	The knowledge of disability issues	0.273	0.103	7.012	1	0.008	1.314
5	Decision-making in the workplace	−0.318	0.123	6.693	1	0.010	0.727
-	Constant	−1.995	0.185	116.570	1	0.000	0.136

**Table 8:** Results of comparison—groups of ambassadors and non-ambassadors.

Variable	Mean ± st.dev. in Non-Ambassadors Group (*N* = 3281)	Mean ± st.dev. in Ambassadors Group (*N* = 928)	*t* Statistic	df	*p*-Value
Inclusion	2.682 ± 0.816	2.151 ± 0.749	18.689	1604.0	***
Discrimination	3.238 ± 0.837	3.253 ± 1.000	−0.427	1316.5	ns
Gains	3.473 ± 0.792	3.875 ± 0.806	−13.474	1472.0	***
Prospects	2.219 ± 0.913	1.242 ± 0.412	46.777	3425.0	***
Situation	2.399 ± 0.659	2.198 ± 0.659	8.191	1491.6	***
Privileges	2.805 ± 0.838	2.820 ± 0.919	−0.451	1392.0	ns

*** *p* < 0.001, ** 0.001 ≤ *p* < 0.01, * 0.01 ≤ *p* < 0.05, ns–non-significant.

## Appendix A

**Table A1:** Attitudes to Disability Scale (ADS) developed by WHOQOL Group [18].

Area	Item
Inclusion	1. People with a disability find it harder than others to make new friends 2. People with a disability have problems getting involved in society 3. People with a disability are a burden on society 4. People with a disability are a burden on their family
Discrimination	5. People often make fun of disabilities 6. People with a disability are easier to take advantage of (exploit or treat badly) compared with other people 7. People tend to become impatient with those with a disability 8. People tend to treat those with a disability as if they have no feelings
Gains	9. Having a disability can make someone a stronger person 10. Having a disability can make someone a wiser person 11. Some people achieve more because of their disability (e.g., they are more successful) 12. People with a disability are more determined than others to reach their goals
Prospects	13. Sex should not be discussed with people with disabilities 14. People should not expect too much from those with a disability 15. People with a disability should not be optimistic (hopeful) about their future 16. People with a disability have less to look forward to than others

**Table A2:** Questionnaire items used to define Ambassador’s attitude [18,25].

No	Question
1	The limitations resulting from disabilities can be effectively compensated by a suitable workplace or its equipment
2	People should not expect too much from those with a disability

**Table A3:** Distribution of the answers to questions used to define Ambassador’s attitude.

People Should Not Expect Too Much from Those with a Disability	The Limitations Resulting from Disabilities Can Be Effectively Compensated by a Suitable Workplace or its Equipment					Total
	1—I Fully Disagree	2	3	4	5—I Fully Agree	
1—I fully disagree	109	64	196	339	928 ^1^	1636
2	22	69	178	361	360	990
3	26	76	353	271	239	965
4	13	36	101	151	100	401
5—I fully agree	20	6	44	40	107	217
Total	190	251	872	1162	1734	4209

^1^ Ambassador of people with disabilities in the workplace.
